# Influence of Mechanical Properties of Steel and CFRP Tapes on the Effectiveness of Strengthening Thin-Walled Beams

**DOI:** 10.3390/ma14092388

**Published:** 2021-05-04

**Authors:** Ilona Szewczak, Patryk Rozylo, Katarzyna Rzeszut

**Affiliations:** 1 Department of Structural Engineering, Faculty of Civil Engineering, Lublin University of Technology, 36 Nadbystrzycka Str, 20-618 Lublin, Poland; 2 Department of Machine Design and Mechatronics, Faculty of Mechanical Engineering, Lublin University of Technology, Nadbystrzycka 36, 20-618 Lublin, Poland; p.rozylo@pollub.pl; 3 Faculty of Civil and Transport Engineering, Institute of Building Engineering, Poznan University of Technology, 5 Marii Skłodowskiej-Curie Str, 60-965 Poznań, Poland; katarzyna.rzeszut@put.poznan.pl

**Keywords:** CFRP tapes, steel tapes, thin-walled cold-formed steel beam, reinforcement method, adhesive

## Abstract

The paper presents a comparison of the effectiveness of strengthening steel thin-walled, cold-formed sigma beams with CFRP tapes and steel tapes. For this purpose, three beams without reinforcement (reference beams) of the “Blachy Pruszyński” type, with a cross-section of ∑200 × 70 × 2 and a span of 280 cm, made of S350GD steel grade, were subjected to laboratory tests in the four-point bending scheme. In the next stage the tests included nine ∑200 × 70 × 2 beams reinforced with Sika CarboDur S512 CFRP tape and six ∑200 × 70 × 2 beams reinforced with steel tape made of S235 steel grade. The length of the reinforcement tapes as well made of steel as well of CFRP was of 175 cm. The location of the tapes within the height of the beams’ cross-section was assumed in three variants, namely placing the tape on the upper or bottom flange and on the web. In the case of beams reinforced with CFRP, three beams were tested for each reinforcement location, and in the case of beams reinforced with steel tapes, two beams were tested for each reinforcement location. SikaDur^®^-30 glue with a thickness of 1.3 mm was used in order to connect steel or CFRP tapes to the beams. The dimensions of the tapes cross-sections in both cases were similar (CFRP tapes: 50 × 1.2 mm, steel tapes: 50 × 1.3 mm). For all types of beams, numerical models were also developed in the Abaqus software. The main aim of this paper was investigation of the influence of mechanical properties of steel or CFRP tapes on the effectiveness of strengthening ∑ beams. For this purpose a comparison of these two solutions with respect to the limitation of displacements and deformations of the beam was performed. The obtained results were considered in the context of the mechanical properties of the materials composing the reinforcement tapes. The tests showed slight differences in the results of strain and displacements obtained for reinforcement made of two different materials. It was also noted that the decisive element was the failure of the joint at the steel-glue interface. Therefore, future studies will pay particular attention to the adhesive layer.

## 1. Introduction

In recent years, CFRP (Carbon Fiber Reinforcement Polymers/Plastics) tapes have found widespread use for reinforcing concrete and masonry structures [1,2]. It became possible, thanks to the introduction of transparent and simplified design procedure rules, to use tapes for this type of construction [1,2]. Currently, there are an increasing number of studies describing research on the use of CFRP tapes to strengthen timber structures [3,4], and even cast iron structural elements [5].

In the case of steel structures, designing their reinforcement often involves the need to modify the static scheme of the structure, increase the cross-section of structural elements or increase the global stiffness of the structure, as it was described by the example of strengthening steel telecommunications towers [6]. However, such a wide scope of works is not always required to strengthen steel structures. When CFRP tapes are used to increase the load-bearing capacity of steel structures made of hot-rolled profiles, many publications confirm the high effectiveness of this method of reinforcement. As an example, it was proved by the study of beams made of an I-section subjected to bending [7,8], box girders [9] or old steel plates [10]. Due to the multitude of published works related to the reinforcement of steel structures made of hot-rolled sections with the use of CFRP tapes, it can be stated that this topic is quite well known. On the other hand, it should be emphasized that in recent years, due to an increasingly high usage of cold-rolled profiles especially as purlins in steel halls, but also as the main load-bearing elements, it becomes necessary to find an effective and quick method of strengthening this type of structure. Recently published articles include research on the reinforcement of steel thin-walled elements using CFRP tapes (for example, axially compressed columns with a square [11,12], circular [13] or C-shaped [14,15] cross-section, or eccentrically compressed columns made of U-profiles [16]). Nevertheless, there are a limited number of publications dedicated to cold-formed, thin-walled steel elements subjected to bending in the field of their reinforcement. Such knowledge can be extremely useful when it is necessary to strengthen steel cold-formed elements used as, for example, purlins or rail in steel hall buildings. It should be emphasized that the motivation to take up this topic was the need to strengthen steel purlins made of cold-formed sigma steel elements (due to snowdrifts) in one of the supermarkets in Poland. Therefore, the authors of this study conducted a study of CFRP reinforced sigma beams [1,17]. Each time, the research showed a satisfactory effect of the use of CFRP tapes on the limitation of strain and displacements of the reinforced elements. Due to the significant cost of composite tape materials, it was decided to conduct analogous tests of sigma beams reinforced with steel tapes [2]. In this study, a comparison of the effectiveness of reinforcement of steel cold-formed sigma beams with CFRP tapes and with steel tapes was presented.

### 1.1. Adhesive Joints

On the basis of the conducted literature review, it can be concluded that the topic of research and analytical calculations of adhesive joints is the subject of work of many researchers. In 1973, in the paper [18], the plastic behaviour of an adhesive was already described using a spring-plastic shear stress model. The author of the paper obtained an equation that represents the maximum rupture stress as a function of the maximum shear stress. The rupture stresses were limited to the elastic range since the internal tensile strength of the laminate is much lower than the rupture strength of typical adhesives.

The authors of the paper [19], referring to the analyses described in [20], developed formulas for determining shear stresses in the plywood and rapture stresses in the CFRP laps under tensile static loading. For the study, they used specimens with CFRP laps on both sides. The following assumptions were made for the investigations: the presence of elastic stress–strain relationships in the adhesive, steel, and CFRP, the absence of slippage between the steel and CFRP, the presence of constant shear stresses across the adhesive thickness, and the presence of a thin plywood layer. In the paper [21], stress and failure analyses were carried out for joints with double-lap adhesive. The stresses obtained from the analytical analyses were compared with the results of numerical tests and it was shown that the simplified analytical solution is sufficient to determine the rapture stresses. Furthermore, in paper [22], a method for determining the shear strength of a joint assuming nonlinear stress–strain behaviour in the plywood was described. 

The authors of many works [23,24,25] are looking for solutions to achieve a permanent reinforcement system and are considering different types of solutions to increase the strength of the adhesive bonded joint. One of these solutions is to reduce the stress in the adhesive layer. In the paper [23], it was found that in a single-lap composite joint, the concentration of shear and rapture stresses occurs at the end of the lap. This phenomenon can be reduced by using an adhesive outflow outside the composite. Finite Element Analyses (FEA) determining the effect of adhesive outflow geometry on the stress state in a single-lap joint are described in [24]. Laboratory studies of the effect of adhesive outflow shape on rapture delay were described in [25,26]. 

The authors of the above-mentioned works observed a satisfactory agreement between the results of laboratory and numerical tests and mathematical analyses of bonded (adhesive) joints. However, the work mentioned above refers to minor lap connections. Nevertheless, it is a motivation to explore the present topic by performing numerical analyses [27,28], taking into account the rupture processes at the adhesive–steel interface.

### 1.2. Reinforcement Methods versus Theory of Thin-Walled Members

The basis for the calculation of stresses in thin-walled cold-formed open steel sections is the issue of thin-walled beam theory. The basic assumption of this theory is the rigid contour hypothesis. This theory takes into account torsion of the bar and deplanation (so called warping), which makes it impossible to apply the classical beam theory also known as Euler–Bernoulli beam theory. A precursor in the field of thin-walled theory was Timoshenko (1961); his work was then developed by Vlasov (1963), who formulated a set of differential equations giving the basis for flexural, torsional or flexural-torsional buckling analysis. In the case of bending sigma beams, loaded in a plane parallel to the web, which do not pass through the shear centre, normal stresses arise due to restrained warping producing the bi-moment next to normal stresses caused by bending. Normal stresses are accompanied by shear stresses caused by restrained warping (Vlasov) distributed evenly across the wall thickness, and shear stresses from free warping (St. Venant). Therefore, the distribution of stresses on the web of a sigma beam is complicated. Thus, starting with the laboratory tests, the authors decided to strengthen the beams in the upper or bottom flange and in web.

The method of reinforcing the beams used in the research characterizes a significant deviation from the classic methods of strengthening the structure. This especially applies to the lack of coincidence of the centres of gravity in the basic and reinforcing sections in the and out of the plane. The proposed method deliberately deviates from this principle due to technological limitations, resulting from access to the reinforced element “in situ” and due to the geometry of the sigma cross-section itself, which has only one axis of symmetry.

Of course, bonding the reinforcement tapes to the flanges each time changes the position of the reinforced cross-section gravity centre in relation to the basic one, and has its impact on the displacements and deformations values. Moreover, the conducted pilot studies showed that beams underwent a global or local form of loss of stability which caused the web deformation. Hence, there was an attempt to strengthen the beams by placing the reinforcing tapes on the web, knowing that it would change the geometrical characteristic of the cross-section to the smallest extent. However, it can significantly counteract excessive web deformation.

## 2. Laboratory Stand

**Figure 1:**
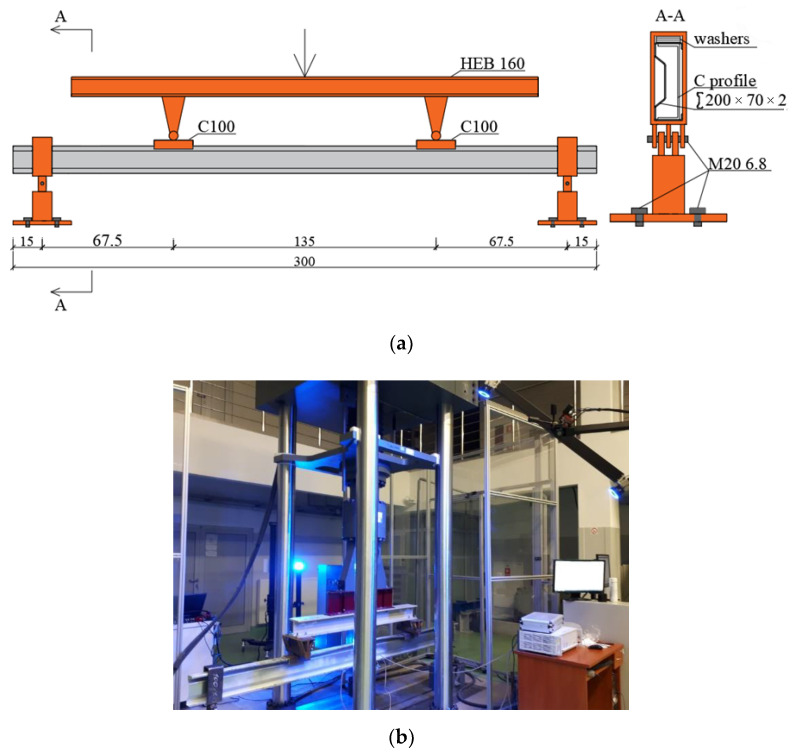
Laboratory stand: (**a**) scheme, and (**b**) photography [2].

Laboratory tests in the four-point bending scheme were applied to beams of the “Blachy Pruszyński” type with the cross-section dimensions of ∑200 × 70 × 2 and the span of 280 cm, made of S350GD steel grade. Two beams were unreinforced (reference beams), nine beams were reinforced with Sika CarboDur S512 CFRP tape and six beams were reinforced with steel tapes of S235 steel grade. The length of the reinforcement tapes was 175 cm. A detailed description of the laboratory tests is presented in [1,2]. The schematic drawing of the laboratory stand is presented in Figure 1a (dimensions are given in cm), while its real form is presented in Figure 1b.

**Figure 2:**
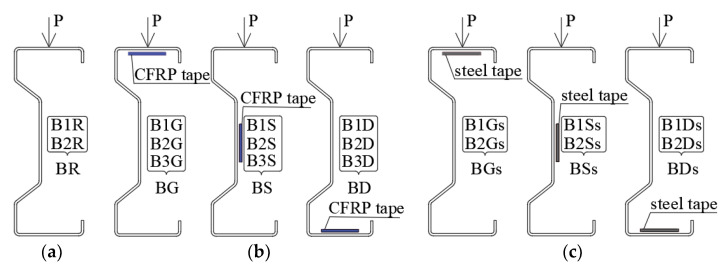
Scope of the tests: (**a**) reference beam, (**b**) CFRP tapes location and sample symbols, and (**c**) steel tapes location and sample symbols.

The beams were reinforced in three variants, placing the tape on the upper and bottom flange or on the web. In the case of beams reinforced with CFRP tapes, three beams were tested for each reinforcement location, and in the case of beams reinforced with steel tapes, two beams for each reinforcement location were tested. To simplify the comparison of the laboratory results of the displacement and strain of beams reinforced with steel or CFRP tapes, it was decided to determine the arithmetic mean value of the results obtained by the samples reinforced in the same way. Therefore, the designations of the beams were unified. For example, the B1R and B2R beams are reference beams, without reinforcement, and the arithmetic mean of the displacement and strain values obtained for these beams are referred to as BR (Figure 2). The same procedure was applied to all other tested beams. It means that BG is the designation of the arithmetic mean value determined for the group of beams reinforced with CFRP tape placed on the upper flange, BS is the group of beams reinforced with CFRP tape on the web and BD is the designation of beams reinforced with CFRP tape placed on the bottom flange. The groups of beams reinforced with steel tape were named similarly (BGs—steel tape placed on the upper flange, BGs—on the web, BDs—on the bottom flange). The location of the steel tapes and CFRP tapes is shown in Figure 2.

SikaDur^®^-30 glue (Sika Poland, Warsaw, Poland) with a thickness of 1.3 mm was used for each joint. The dimensions of the tapes cross-sections were similar for both tapes: CFRP—50 × 1.2 mm and the steel—50 × 1.3 mm. The basic mechanical properties of the materials used in the study are presented in Table 1.

Note that the reinforced beam consists of three different materials, namely cold-formed steel material, tape material and glue. That is why the considered beams are the composite elements made of structural steel S350GD grade, characterized by E = 201.8 GPa, ν = 0.282 and of reinforcement with CFRP tapes of Sika Carbodur S512 tape with E = 165 GPa and ν = 0.308 or steel tape is made of the S235 steel grade with the Yield strength lower by nearly half, and with similar values of E and ν in relation to the profile steel grade. The main difference between the used tapes is in their stiffness, which can have a significant impact on the cooperation of the tape with the steel beam. Steel thin-walled beams under load may undergo significant local and global deformations, therefore a tape with lower stiffness may prove to be a more advantageous solution; this especially is the case, given that the lower stiffness of CFRP tapes does not mean that the material is weaker. It should be added that CFRP tape, as a composite material, has many advantageous features, such as: over ten times higher tensile strength in the fibres’ direction compared to typical steel grade, and very high fatigue strength and resistance to aggressive factors in term of corrosion. Another feature that distinguishes steel tape from CFRP is the elongation percentage, which in the case of CFRP tape is 1.8%, and for steel tape is 22.5%. In both cases, the tapes are connected to the beam using SikaDur-30 glue, which has strength properties that are significantly different from other elements of the reinforced beam.

## 3. Laboratory Results

**Figure 3:**
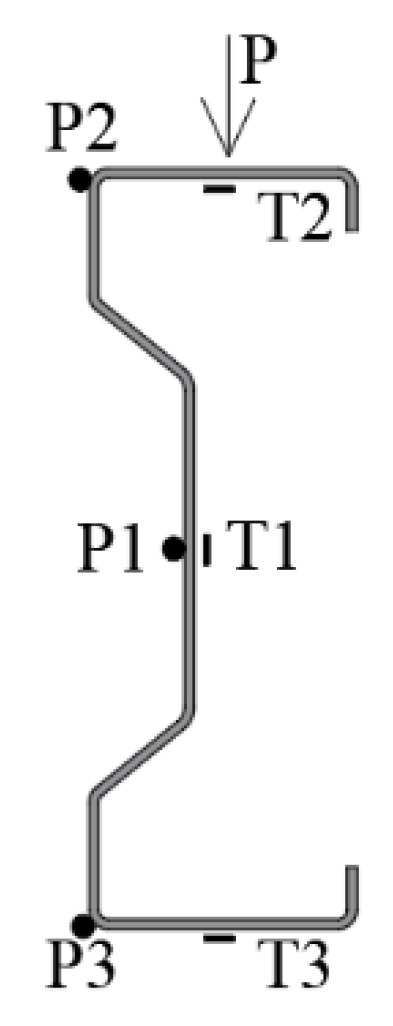
Location of displacement measurement points (P1, P2, P3) and electrofusion strain gauges in the middle of the beam span.

During the laboratory tests, the strains were measured using the electrofusion strain gauges at T1, T2 and T3 points, and the displacements at P1, P2 and P3 points were measured using the Aramis and Tritop optical measuring system. All displacement and strain measurement points were placed in the middle of the beam span. The location of the points in the cross-section is shown in Figure 3.

### 3.1. Strain Analysis

**Figure 4:**
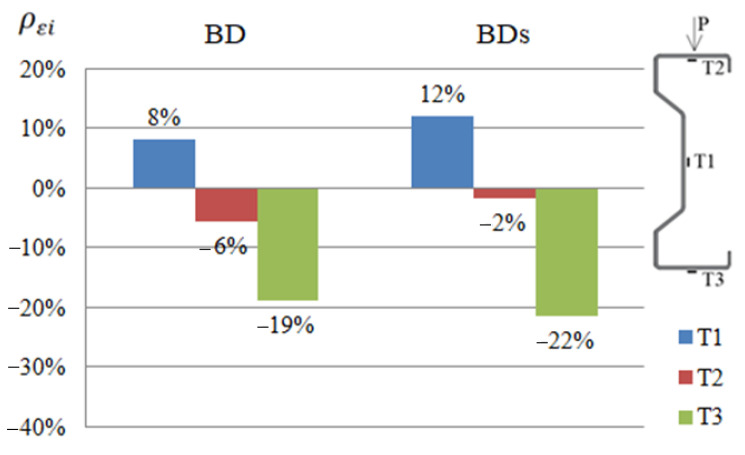
Percentage limits of strains $\rho_{\varepsilon i}$ at T1, T2, T3 points of the tested beams reinforced with steel or CFRP tapes on the bottom flange, in relation to the reference beams at a load of 25 kN.

**Figure 5:**
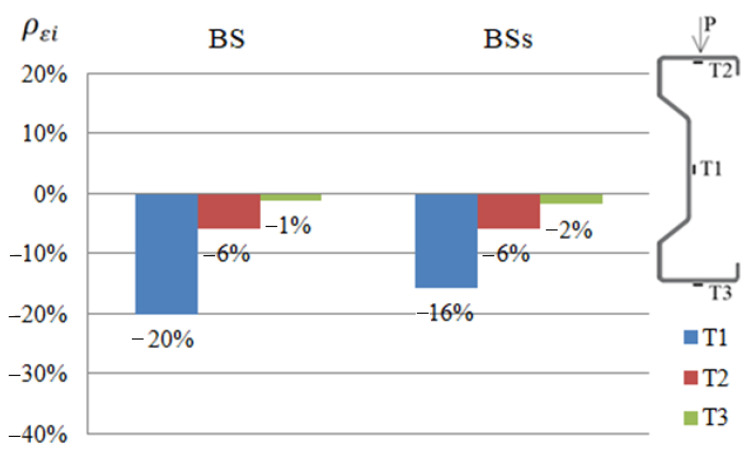
Percentage limits of strains $\rho_{\varepsilon i}$ at T1, T2, T3 points of the tested beams reinforced with steel or CFRP tapes on the web, in relation to the reference beams at a load of 25 kN.

**Figure 6:**
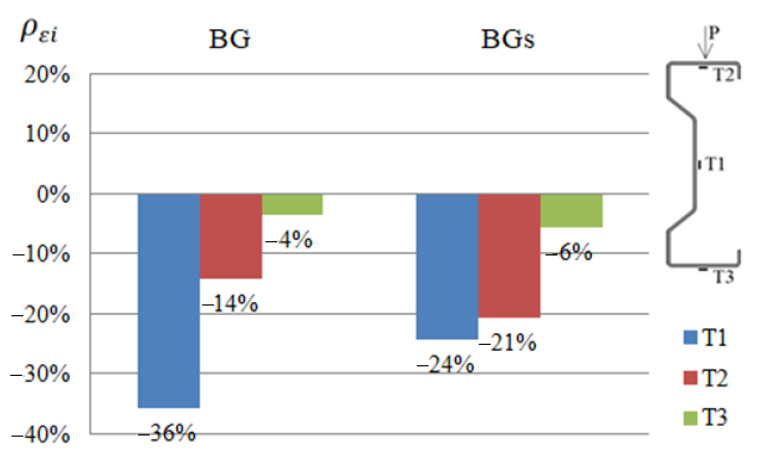
Percentage limits of strains $\rho_{\varepsilon i}$ at T1, T2, T3 points of the tested beams reinforced with steel or CFRP tapes on the upper flange, in relation to the reference beams at a load of 25 kN.

In order to compare the strain results of beams reinforced with CFRP or steel tape, appropriate diagrams were prepared (Figure 4, Figure 5 and Figure 6). The symbols BG, BD and BS given in the diagrams represent groups of beams reinforced with CFRP tape placed on the upper or bottom flange and on the web, respectively. Similarly, symbols BGs, BDs and BSs are groups of beams reinforced with a steel tape placed on the upper or bottom flange and on the web. The presented results were determined at a load of 25 kN, considered as a failure load, due to the fact that both steel and CFRP tapes were debonded after exceeding this load level.

For each of the tested beams, the effect of applying a tape reinforcement (steel or CFRP) on the reduction in strain was determined, using the relationship:

(1) $$\rho_{\varepsilon i}=\left(\frac{\varepsilon_{i}}{\varepsilon_{ref}}-1\right)\times 100\%$$

where: $\rho_{\varepsilon i}$—reduction or increase in strain of a given sample expressed as a percentage, $\varepsilon_{i}$—strain of a given sample, and $\varepsilon_{ref}$—arithmetic mean value of the strain of two reference beams.

Negative values indicate a reduction of strain. Percentage values of increase or reduction of strains given in the graphs constitute the arithmetic mean determined from the results obtained by the beams belonging to a particular group. For example, the B1D, B2D and B3D beams belong to the BD group. 

Based on the presented summary diagrams, it was found that in order to limit the strain of the web (in the place of the T1 strain gauge), it is more advantageous to use a reinforcement made of CFRP tapes. In the case of beams reinforced in the upper flange, it allowed reducing deformations by 12% more than in the case of beams reinforced with steel tape. In order to limit the strain in the compressed flange (strain gauge T2) in diagram 5, the obtained results are the same, but diagram 6 indicates that the reinforcement may be more advantageous with steel tapes (deformation reduction by 7% compared to CFRP tapes). In case of the tensioned flange (strain gauge T3) it can be concluded that reinforcement with the use of steel tapes is more effective. The use of steel tapes at each location reduced the deformation recorded by the T3 strain gauge to a greater extent than the use of CFRP tapes. It is worth noting, however, that at this point the percentage limitation of displacements obtained for the beam reinforced with steel and CFRP tapes is quite similar. Moreover, the results of strain of individual beams reinforced with a steel tape were characterized by a fairly large dispersion of the results [2], therefore, based on a limited number of samples, no unambiguous conclusions should be drawn. 

### 3.2. Displacement Analysis

**Figure 7:**
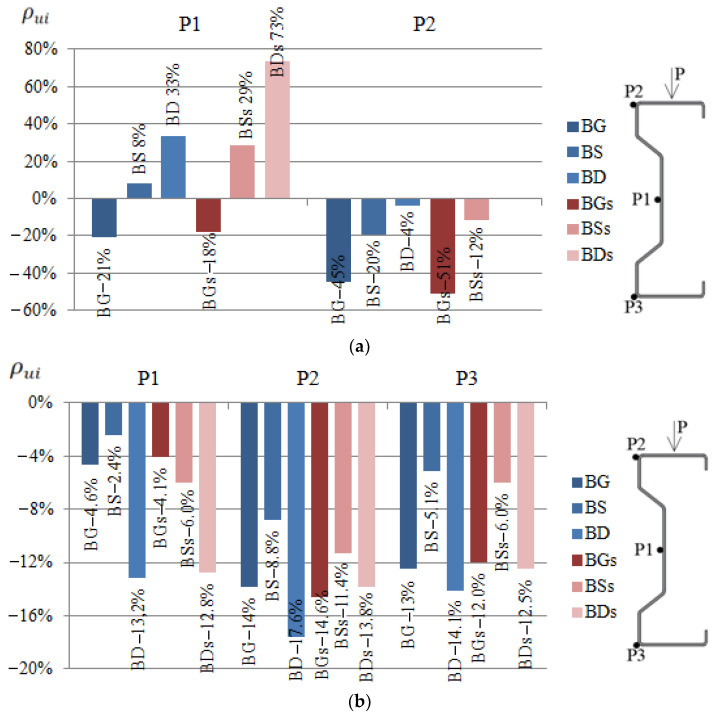
Percentage reduction of displacements $\rho_{ui}$ at P1, P2 i P3 points of the tested beams reinforced with steel or CFRP tapes on: (**a**) the horizontal, in the direction perpendicular to the longitudinal axis of the beam, (**b**) the vertical, in relation to the reference beams at a load of 25 kN.

The comparison of the results of displacement of beams reinforced with steel or CFRP tapes was prepared in a similar way to strain analysis (Figure 7). The values given in the graphs were calculated by determining the arithmetic mean of the value determined from Equation (2) for all samples strengthened in the same way, thus belonging to one group.

(2) $$\rho_{ui}=\left(\frac{u_{i}}{u_{ref}}-1\right)\times 100\%$$

where: $\rho_{ui}$—percent change in displacement of the ith sample, $u_{i}$—displacement of the ith sample, and $u_{ref}$—displacement of reference beam.

During the laboratory test, it was observed that with a load of 25 kN, both beams reinforced with steel or CFRP tapes were deflected by an average of more than 11 mm and moved out of the plane by more than 10 mm. The exact values of the displacements were presented in [1,2].

Based on obtained results regarding the displacement of beams reinforced with steel or CFRP tape, it was found that in order to limit vertical displacements, the reinforcement should be placed in the bottom flange of the beams, that is, the tensioned one. However, if the horizontal displacement should be limited, the upper (compressed) flange should be strengthened using CFRP tapes. In the case of using the reinforcement in the upper flange, the horizontal displacements at points P1 and P2 in relation to the reference beams were reduced by 21% and 45%, respectively, in the case of reinforcement with CFRP tape, and by 18% and 51%, respectively, in the case of using steel tape (Figure 7a). In the case of using the reinforcement in the bottom flange, the vertical displacement at points P1, P2 and P3 was limited in relation to the beams without reinforcement by 13.2%, 17.6% and 14.1%, respectively, for the CFRP tapes, and 12.8%, 13.8% and 12.5%, respectively, for the steel tapes.

Reinforcement made of CFRP tapes allowed reducing displacements to a greater extent, which speaks in favour of this type of reinforcement. However, in order to unambiguously formulate conclusions, it would be necessary to conduct tests on a larger number of samples.

## 4. Numerical Analyses

Regarding the numerical simulations, all analyses using the finite element method (in Abaqus^®^ software) were conducted. A detailed description of the numerical models was presented in papers [1,2]. In the case of conducted investigations, reflecting the laboratory tests, the prepared numerical model of the steel beam—sigma shape, is made using shell finite elements—known as S4R. Most often thin-walled shell elements are prepared using S8R shell elements. In the presented work, the results obtained for S4R shell elements with linear shape function and reduced integration were more similar to the experimental results than for S8R elements [1,2]. The above-mentioned shell finite elements had 6 degrees of freedom—both three translational and rotational, in each of four nodes, per one finite element. In addition, the numerical studies used special steel washers, which were mainly used to carry the load from the press to the beams and steel support clamps. Construction of steel washers enabled the creation of the fork support; they were prepared using non-deformable shell elements (known as R3D4). The R3D4 shell elements had 3 degree of freedom—only translational, in each of the 4 nodes, per one finite element. In order to reduce the local deformation, the C-shape profiles were placed inside the beam (near structure supports). The C-profiles were made using a S4R shell finite elements, the same type of finite elements as used for the main sigma beam structure (thickness corresponding to the real profile). In the presented work, a structural type of mesh was used (regardless of the type of finite elements). Based on the stress–strain (σ-ε) characteristic obtained in laboratory test, the elastic-plastic bilinear material model including strain hardening was adopted for the beam. The applied material model was characterized by parameters: Young’s modulus (201.8 GPa), Poisson’s ratio (0.282), and Yield strength (418.5 MPa). The material parameters adopted in FEM numerical model are compatible with the data presented in Table 1. A slightly lower value was used in the case of Young’s modulus adopted in the study, than for the typical S350 GD steel grade; this is conditioned on the fact that the specimens are made of galvanised steel.

**Figure 8:**
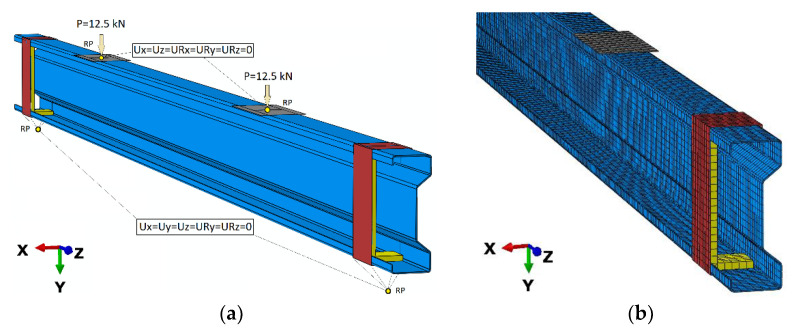
Numerical model: (**a**) model with boundary condition, and (**b**) discrete model.

Regarding the discrete models of washers and steel clamps, special reference points were defined. In the above-mentioned reference points, the boundary conditions were defined—Figure 8. Additionally, the load was defined as two independent forces (equal in value P = 12.5 kN) at previously defined reference points. With reference to the contact interactions, the contact relations, both in the normal and tangential direction (without friction coefficient) were defined. The above-mentioned contact relations were implemented between the supports and the beam, between C-profile sections and the beam and also between washers and the beam (Figure 8). The boundary conditions defined within the numerical simulations, corresponded to the experimental boundary conditions. The bottom supports were fork supports, where only rotation was possible to allow bending (relative to the X-axis—as shown in Figure 8). The upper loading elements were a reproduction of the experimental study, where only displacement in the downward direction (relative to the Y-axis—as also shown in Figure 8) was possible.

The finite element model included 18,312 nodes, 17,713 finite elements (of which 16,273 constituted deformable shell elements—for main steel beam and C-profiles, 1440 elements constituted non-deformable linear shell finite elements of supports). Regarding the material model of steel tapes, the bilinear, elastic-plastic model was adopted (Table 1). The material model of CFRP tapes had special orthotropic properties (E_1_ = 142 GPa, E_2_ = 8 GPa, ν_12_ = 0.308 and G_12_ = G_23_ = G_13_ = 4.5 GPa). The above-mentioned CFRP tapes were prepared using shell finite elements combined to the beam using TIE relations. Firstly, the bare beam (denoted as BRa) finite element model was prepared, for which verification based on the experimental tests was carried out. After obtaining agreement of results for the bare beam, 6 subsequent finite element models were developed, respectively: BGsa with steel tape placed in the upper flange (analogously to the BGs), BDsa with steel tape in the bottom flange (like BDs), the BSsa in which steel tape was placed in the web (corresponding to BSs), BGa with CFRP tape placed in the upper flange (analogously to the BG), BDa with CFRP tape in the bottom flange (like BD) and the BSa with CFRP tape in the web (corresponding to BS). 

**Figure 9:**
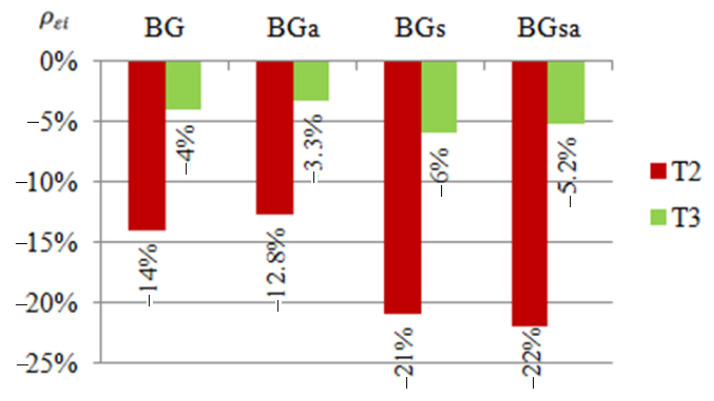
Percentage limits of deformation of reinforced beams in the upper flange.

**Figure 10:**
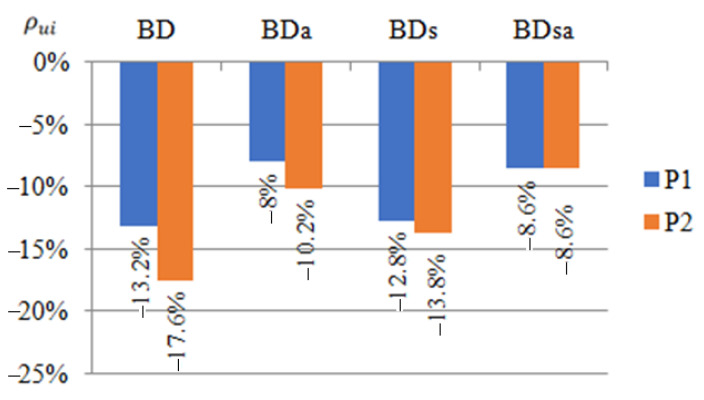
Percentage limits of vertical displacement of reinforced beams in the bottom flange.

The numerical analysis of strains of the steel beam was limited to the assessment of results in places of electrofusion strain gauges denoted as T2 and T3 that were placed during experimental tests. The strain level readout was confronted with strain Max. In-Plane Principal (Abs) from numerical simulations was performed in the Abaqus program. These are strain components analysing in the longitudinal direction (of the steel beam) in the plane of the particular walls of construction. Regarding the numerical simulations, the horizontal and vertical displacements of the beams were also measured at the points corresponding to the experimental tests. Example results, elaborated according to Equations (1) and (2) demonstrate the comparison of numerical to laboratory test results, and are shown in the following figures (Figure 9 and Figure 10). The numerical results obtained confirm the trend coming from the laboratory tests and confirm the agreement of the numerical analyses with the laboratory tests. The discrepancies may result from the fact that the displacement and deformation results of the beams tested in the laboratory are the arithmetic means of several specimens. In order to improve the quality of the results, laboratory tests should be conducted on a larger number of specimens. 

Both in the case of beams reinforced with CFRP and steel tape, the beam deformation was consistent between laboratory and numerical tests. Examples of deformation forms are presented in [1,2].

## 5. Final Remarks

In this paper, the analysis of the effectiveness of reinforcement of thin-walled cold-formed sigma beams using steel or CFRP tapes, with particular emphasis on the impact of their location, and on the field of strain and displacement was presented. The wide range of research studies were carried out including pilot and target laboratory tests and corresponding numerical analysis. Bearing in mind that it is very difficult in the laboratory to accurately reproduce the conditions existing in real structures subjected to reinforcement, that is, under preloading, each time unreinforced (reference) beams were tested; based on those conditions, the effectiveness of individual types of reinforcement was determined. As is commonly known, the existence of an initial state of stress can significantly affect the effectiveness of the performed reinforcement. Therefore, in engineering practice, it is recommended to unload the structure subject to strengthening as much as possible. Considering the above statements, it was assumed in this paper that the performance of research on the effectiveness of reinforcement of beams without preloading, but with respect to reference beams, can be considered reliable and can provide important information useful for the design of reinforcement of these types of elements.

It is worth recalling that on the basis of various laboratory tests of steel elements reinforced with CFRP tapes, the following forms of damage were observed: adhesive failure on the CFRP—adhesive interface, the so-called debonding, delamination inside the composite (CFRP delamination) or CFRP tensile failure. In the study presented in this paper, failure was observed at the adhesive-steel interface, at a load level of 25 kN which is described and illustrated in detail in [1,2]. Thus, it turned out that in the case of reinforcing a steel cold-formed beam, the weakest element was not the reinforcing tape, but the adhesive. This fact is an additional motivation to undertake further research on glued joints.

## 6. Conclusions

Comparing the results obtained for cold-formed sigma beams reinforced with steel or CFRP tapes, it was found that in order to limit displacements (both in the vertical and horizontal directions) and strain in the beam web, the use of CFRP tapes is more advantageous. On the other hand, in order to limit the strain in both flanges, the use of steel tapes was found to be more effective. It should be emphasized that in the case of displacements, the differences in the results achieved for beams reinforced with steel tape and CFRP tape did not exceed 6%, which is a small value. Moreover, the works [1,2] indicated the influence of the reinforcement location on cross-sectional geometric characteristic and obtained results. For example, the use of the CFRP tape in the lower or upper flange of the beam causes an increase in the moment of inertia with respect to the y axis by 18%, and with respect to the z axis by nearly 9%, and a change in the position of the centre of gravity of the section by 1.26 mm horizontally and by 13.6 mm vertically. At the same time, placing the tape in the lower flange allows reducing the vertical displacement of the beam by 17.6% and the horizontal displacement by 4%, and the use of CFRP tape in the upper flange allows reducing the vertical displacement of the beam by 14% and the horizontal displacement by up to 45%.

An interesting fact is that placing the CFRP tape on the web changes the moments of inertia with respect to both axes by less than 0.5% and causes a shift of the centre of gravity by only 0.32 mm in the horizontal direction, and at the same time reduces vertical displacement by 8.8% and horizontal displacement by up to 20%; this seems to be a very promising result.

In addition, based on set of numerical models developed in the Abaqus program for all stages of laboratory tests, the influence of the reinforcement application on the reduction of strains and displacements of sigma beams observed in laboratory tests has been confirmed. However, the obtained results concerning the reinforcement of the beam with CFRP or steel tapes do not differ enough to clearly indicate the best reinforcing material. It should be remembered that making the reinforcement with steel tapes increases the weight of the element, but the cost of the steel tapes is much lower than CFRP tapes. Therefore, when starting to design the reinforcement of a cold-formed steel element, it is necessary to carefully analyse the advantages and disadvantages of the reinforcement methods. Considering both, it provides designers the opportunity to undertake a justified decision on the type of reinforcement in terms of strength and economy issues. It should also be emphasized that in designing the reinforcement of a cold-formed steel element with tapes (steel or CFRP), the key is to determine the strength of the adhesive joint. This issue will become the subject of further research conducted by the authors of this paper.

## Figures and Tables

**Table 1:** Mechanical properties of tested elements materials.

Mechanical Properties	Steel Beam S350GD	CFRP Tape Sika Carbodur S512	Steel Tape S235	SikaDur-30
Young’s modulus	201.8 GPa	165 GPa	210 GPa	-
Poisson’s ratio	0.282	0.308	0.300	-
Yield strength	418.5 MPa	-	235.0 MPa	-
Elongation	-	1.8%	22.5%	-
minimum compressive strength	-	-	-	75 MPa after 7 days
modulus of elasticity under compression	-	-	-	9600 MPa
minimum tensile strength after 7 days	-	-	-	26 MPa
deboning strength from steel after 7 days	-	-	-	21 MPa
shear strength	-	-	-	16 MPa
Shrinkage	-	-	-	0.04%

## Data Availability

Data is contained within the article.
